# Laparoscopic Hernia Sac Transection With Intracorporeal Ligation: A Comparison With Open Hernia Repair in the Pediatric Age Group

**DOI:** 10.7759/cureus.75633

**Published:** 2024-12-13

**Authors:** Nancy Thakur, Rakesh K Tripathi, Pawan K Singh, Shraddha Verma, Rajesh K Maurya, Ayushya Gupta

**Affiliations:** 1 Department of General Surgery, Ganesh Shankar Vidhyarthi Memorial Medical College, Kanpur, IND

**Keywords:** comparative outcomes in hernia repair, laparoscopic herniotomy, minimally invasive surgery in children, open herniotomy, paediatric inguinal hernia

## Abstract

Background and objective

Inguinal hernia in children results from a failure of the processus vaginalis (PV) to close, leading to herniation. Surgical repair is necessary to prevent complications in this patient population. This study aimed to compare the outcomes between laparoscopic herniotomy (LH) and open herniotomy (OH) in pediatric patients with inguinal hernia.

Methods

This prospective study included 80 children diagnosed with inguinal hernia between January 2023 and June 2024. Patients aged 1-12 years were classified into two groups: 38 underwent LH, and 42 received OH. Postoperative outcomes, including operative time, pain, hospital stay, and complications, were evaluated. Statistical analysis was performed using descriptive statistics, with a p-value <0.05 considered significant. The t-test, chi-square test, and arithmetic mean with standard deviation (SD) were used for data analysis.

Results

The mean operative time was significantly longer for LH (97.89 ±18.2 minutes) compared to OH (31.14 ±8.3 minutes) (p<0.001). However, pain assessment revealed no significant difference between the groups (p=0.776). Hospital stay was longer for LH patients, who were discharged on day two, compared to day one for OH patients (p=0.001). No significant differences were observed in surgical site infection (SSI) rates (p=0.090) or return to school (p=0.857).

Conclusions

Laparoscopic and open hernia repair are comparable in terms of postoperative pain, SSI rates, and return to normal activities. However, OH offers advantages in terms of reduced operative time and shorter hospital stays. Further studies, including randomized controlled trials, are needed to exclude selection bias and provide deeper insights into the topic.

## Introduction

Inguinal hernia in children is a developmental issue linked to the descent of the gonads. During fetal development, the processus vaginalis (PV) forms at around the 12th week and typically closes by the 36th-40th week, except for the tunica vaginalis. In some newborns, incomplete closure of the PV leaves a patent processus vaginalis (PPV), causing abdominal contents to herniate through the inguinal canal [1,2]. The incidence of inguinal hernia in the pediatric population ranges from 1.0% to 5.0%, and it is significantly higher in premature infants [3].

Surgical intervention is advised for all children diagnosed with an inguinal hernia due to the risk of incarceration, intestinal obstruction, and strangulation [4]. The traditional treatment involves an open incision called open herniotomy (OH). Although laparoscopic herniotomy (LH) has been around for over two decades, there is still some debate over whether it is the preferred method of inguinal hernia repair. Conventional open repair of inguinal hernia is considered the gold standard treatment for pediatric inguinal hernia (PIH) due to the unremarkable scar it leaves behind and lower recurrence rates [5]. However, over the last two decades, advances in pediatric minimally invasive surgery (MIS) have made laparoscopy increasingly popular for managing PIH [6]. In a 2014 survey, 83% of the respondents preferred the open approach [7]. Previous systematic reviews in 2014 and 2019 comparing LH and OH did not reach a consensus as to which technique is superior [8-10]. In light of this, we performed a comparative analysis of laparoscopic hernia repair and open hernia repair in the pediatric age group.

## Materials and methods

Study design

This prospective study was conducted in the Department of Surgery, Ganesh Shankar Vidhyarthi Memorial (GSVM) Medical College, Kanpur, over 18 months from January 2023 to June 2024. The study involved 80 pediatric patients diagnosed with inguinal hernia. 

Study population

A total of 80 pediatric patients, aged 1-12 years, diagnosed with inguinal hernias were included in the study.

Inclusion and Exclusion Criteria

Pediatric patients aged 1-12 years with inguinal hernias were eligible for inclusion. Patients with hydrocele, obstructed or irreducible hernias, recurrent hernias, or those older than 12 years were excluded from the study.

Sampling technique and sample size 

The study used purposive convenient sampling. Based on sample size estimation for nominal and ordinal data with proportions (p1=0.4, p2=0.2) and parameters of 95% confidence interval (CI) and 80% power, the minimum sample size was calculated to be 79. A total of 80 patients were enrolled.

Ethical considerations

The study adhered to ethical guidelines, with approval obtained from the Ethics Committee (Human) EC/113/April/2023 of GSVM Medical College, Kanpur, Uttar Pradesh, India. Informed consent was obtained from all participants, ensuring their autonomy in deciding to participate or withdraw without compromising clinical benefits. Strict confidentiality was maintained regarding patient records throughout the study.

Study methodology

Data were collected in both local and English languages to ensure clear communication. Clinical data were complemented by diagnostic and treatment-related information. Two operative techniques - laparoscopic hernia repair and open herniotomy - were utilized, and informed consent was obtained for the chosen surgical approach.

Laparoscopic Hernia Repair

This was performed under general anesthesia in the supine position. The pneumoperitoneum was established using the Hasson cannula or Veress needle, with a 5-mm telescope placed in the infraumbilical region. The diagnosis was confirmed, and two 3-mm ports were introduced laterally below the umbilicus. The PV was excised, ensuring separation from the vas deferens and vessels. The internal inguinal ring was closed by purse string suture laparoscopically, avoiding injury to surrounding structures.

Open Herniotomy

An incision was made in the inguinal crease, and dissection proceeded to expose Scarpa’s fascia. The external oblique aponeurosis was incised, and the space between the internal and external oblique layers was dissected. The spermatic cord was mobilized, and the hernia sac was ligated near the deep inguinal ring using a vicryl suture. Closure of the external oblique aponeurosis and skin was performed.

Postoperative assessments

Pain levels were measured using the Wong-Baker Faces Pain Rating Scale, ranging from 0 (no pain) to 10 (severe pain) (Table 1), and surgical site infection (SSI) was graded using the Southampton Wound Scoring System, ranging from Grade 0 (normal healing) to Grade 5 (deep or severe infection) (Table 2).

**Table 1:** Wong-Baker Faces Pain Rating Scale

Grade	Pain level	Description	Faces
0	No pain	Feeling no pain at all	😃
2	Mild pain	Slight pain, easily tolerable	😊
4	Moderate pain	Pain is present but manageable	😐
6	Severe pain	Pain limits some activities	😟
8	Very severe pain	Pain dominates the senses, difficult to focus	😢
10	Worst pain	Unbearable pain, requires intervention	😭

**Table 2:** Southampton Wound Grading System

Grade	Appearance
0	Normal healing
1	Normal healing with mild bruising or erythema
2	Erythema plus other signs of inflammation, but no discharge
3	Clear or serous discharge, with or without erythema or other signs of inflammation
4	Pus discharge (purulent) with or without wound dehiscence (separation of wound edges)
5	Deep or severe wound infection requiring surgical intervention, such as drainage or debridement

Data collection

Data on demographic, clinical, and surgical parameters were collected using a pre-designed proforma. Outcomes such as operative time, length of hospital stay, postoperative pain, SSI, and return to school were evaluated.

Data analysis

Collected data were entered into Microsoft Excel and analyzed using SPSS Statistics version 23.0 for Windows (IBM Corp., Armonk, NY). Quantitative variables were expressed as mean ± standard deviation (SD), while qualitative data were presented as frequencies and percentages. Parametric (t-tests) and non-parametric tests (chi-square test) were applied as appropriate. Analysis of variance (ANOVA) was used for intra-group variations over time. A p-value <0.05 was considered statistically significant.

Quality control

Measures were implemented to ensure data completeness and consistency. Expert opinions and reputable literature guided the selection of clinical parameters to ensure the validity and reliability of data collection methods.

## Results

The study assessed pediatric laparoscopic hernia repair compared to open hernia repair. Among 80 participants, 38 underwent laparoscopic surgery, and 42 underwent OH. The mean ages of the patients were 4.03 ±3.3 years (laparoscopic) and 4.63 ±2.7 years (open). Both groups had a male predominance, and the right inguinal hernia was the most common variant (Table 3).

**Table 3:** Distribution of patients based on demographics and diagnosis in both groups ^*^The chi-square test was used for age group, gender, and diagnosis, while the independent samples t-test was used for mean age; ^**^p<0.05 was considered statistically significant

Parameter	Laparoscopic herniotomy (n=38)	Open herniotomy (n=42)	P-value^*^
Age group, years, n (%)			
1-5	30 (78.9%)	28 (66.7%)	0.040^**^
6-10	4 (10.5%)	13 (31.0%)	
10-12	4 (10.5%)	1 (2.4%)	
Mean age ±SD	4.03 ±3.3	4.63 ±2.7	0.386
Gender, n (%)			
Male	33 (86.8%)	39 (92.9%)	0.301
Female	5 (13.2%)	3 (7.1%)	
Diagnosis, n (%)			
Left inguinal hernia	12 (31.6%)	16 (38.1%)	0.830
Right inguinal hernia	21 (55.3%)	21 (50.0%)	
Bilateral inguinal hernia	5 (13.2%)	5 (11.9%)	

The mean operative time was significantly higher for laparoscopy (97.89 ±18.2 minutes) than for open surgery (31.14 ±8.3 minutes) (p<0.001) (Table 4).

**Table 4:** Mean operative time among studied patients in both groups ^*^Independent samples t-test was used for operative time; ^**^p<0.05 was considered statistically significant

Operative time, minutes	Laparoscopic herniotomy (n=38)	Open herniotomy (n=42)	P-value*
Mean ±SD	97.89 ±18.2	31.14 ±8.3	<0.001**

Postoperative pain was assessed using the Wong-Baker Faces Pain Rating Scale 48 hours after surgery. In the laparoscopic group, four patients (10.5%) had grade 2 and one patient (2.6%) had grade 4 pain, whereas in OH, three patients (7.1%) had grade 2 pain and two patients (4.8%) had grade 4 pain (p=0.776). The difference was statistically not significant. Hospital stay was longer for laparoscopy, with patients discharged on day two compared to day one for open surgery (p<0.001). There was no SSI in the laparoscopic group, while four patients (9.5%) had grade 1 SSI and one patient (2.4%) had grade 3 SSI in the open group (p=0.09). Return to school in the laparoscopic group was 10 days in 14 patients (36.8%) and 14 days in eight patients (21.1%); there were 16 (42.1%) non-school-going patients in this group. In the OH group, 15 patients (35.7%) and 11 patients (26.2%) returned to school after 10 days and 14 days respectively; this group also had 16 (38.1%) non-school-going patients (p=0.857) (Table 5). No recurrence was seen in either group on follow-up (duration ranging from one month to 18 months).

**Table 5:** Postoperative assessment of the patients in both groups ^*^The Chi-square test was used for postoperative pain, surgical site infection, and return to school, while Fisher’s exact test was used for the duration of hospital stay; ^**^p<0.05 was considered statistically significant

Parameter	Laparoscopic herniotomy (n=38), n (%)	Open herniotomy (n=42), n (%)	P-value*
Postoperative pain (after 48 hours)			
Grade 0	33 (86.8%)	37 (88.1%)	0.776
Grade 2	4 (10.5%)	3 (7.1%)	
Grade 4	1 (2.6%)	2 (4.8%)	
Surgical site infection (Southampton score)			
Grade 0	38 (100.0%)	37 (88.1%)	0.090
Grade 1	0 (0.0%)	4 (9.5%)	
Grade 3	0 (0.0%)	1 (2.4%)	
Duration of hospital stay (days)			
1	0 (0.0%)	42 (100.0%)	<0.001**
2	38 (100.0%)	0 (0.0%)	
Return to school			
10 days	14 (36.8%)	15 (35.7%)	0.857
14 days	8 (21.1%)	11 (26.2%)	
Non-school-going	16 (42.1%)	16 (38.1%)	

## Discussion

The management of PIH has advanced significantly, with laparoscopic techniques increasingly being considered a viable alternative to conventional OH. This study evaluated and compared outcomes between LH and OH, focusing on operative time, postoperative pain, hospital stay, SSI, and return to school. The demographic characteristics of the two groups were comparable, with a mean age of 4.03 ±3.3 years in the LH group and 4.63 ±2.7 years in the OH group. Male predominance was noted in both groups, with 86.8% in the LH group and 92.9% in the OH group. These findings align with Bharathi et al., who reported that males are disproportionately affected by inguinal hernias due to embryological factors such as the incomplete closure of PV [5]. Additionally, the right inguinal hernia was more common in both groups. This right-sided predominance is attributed to the later closure of the right PV during embryogenesis compared to the left side [5,11].

Operative time was significantly longer for LH (97.89 ±18.2 minutes) compared to OH (31.14 ±8.3 minutes). This difference is consistent with findings by Shalaby et al., who noted that the initial learning curve associated with laparoscopic techniques led to extended operative times [11]. However, Chan et al., Lobe et al., and Liu et al. highlighted that with increasing surgeon experience and the adoption of technical refinements, operative times for LH tend to decrease significantly, sometimes matching those of OH [12-14]. In our study, operative times for LH decreased to around 60 minutes in later cases, underscoring the importance of surgeon experience in improving efficiency. This emphasizes the need for dedicated training programs in laparoscopic techniques to overcome this learning curve.

Postoperative complications were minimal in both groups, with no complications reported in the LH group and only one case of SSI documented in the OH group; this contrasts with the findings in the study by Kantor et al., where the LH group had more cases of SSI [9]. However, this finding corroborates that of Zhao et al., who observed significantly lower SSI rates and major complications in LH compared to OH [15]. Esposito et al. also emphasized that LH minimizes tissue trauma, which likely reduces the risk of SSIs and hematomas [10]. These results underscore the safety of LH in pediatric populations, particularly for reducing complications associated with traditional open techniques.

Pain levels between the two groups were comparable. While slightly more patients in the LH group reported grade 2 pain, this difference was not statistically significant. In a similar vein, Bharathi et al. have reported that postoperative pain perception did not differ significantly between LH and OH [5]. Recovery times were also comparable, with the LH group showing a slightly quicker return to normal activities, such as school. Koivusalo et al. found similar recovery rates between the two techniques, attributing the results to effective postoperative pain management in both groups [16]. Interestingly, Boo et al. reported that LH facilitates faster recovery due to reduced tissue manipulation and minimal scarring, factors that can enhance the quality of life for pediatric patients [17].

The duration of hospital stay was longer for the LH group, with all patients discharged on the second day compared to day one for OH patients, which was attributed to cautious postoperative monitoring. However, with the advent of refined laparoscopic techniques, as described by Shalaby et al., the hospital stay for LH can be reduced to match that of OH, which has significant implications for resource utilization and patient turnover [11].

No hernia recurrences were observed in either group during the follow-up period (1-12 months). This result is consistent with findings from Dreuning et al., who reported similar recurrence rates between the two techniques when performed by experienced surgeons [18]. The low recurrence rates in both techniques highlight the efficacy of the current pediatric hernia repair, irrespective of the approach.

Limitations

While LH offers several advantages, its widespread adoption is hindered by the longer operative times associated with the learning curve. Additionally, the slightly higher costs of laparoscopic equipment and training pose challenges, especially in resource-limited settings. The results of this study are limited by the small sample size and relatively short follow-up period, which may have underestimated recurrence rates and long-term complications. Future studies should focus on randomized control trials to remove selection bias and ensure longer follow-up durations to validate these findings.

## Conclusions

This comparative study highlights the relative merits and limitations of LH and OH in PIH repair. Both approaches demonstrated comparable outcomes in terms of postoperative pain, SSI rates, and time to resumption of normal activities. However, OH offered significant advantages in reduced operative time and shorter hospital stays, making it more efficient in certain contexts. Despite the prolonged learning curve associated with LH, its minimally invasive nature underscores its potential as an effective alternative to OH. Further research, particularly randomized controlled trials with larger sample sizes and extended follow-up periods, is essential to validate these findings and address limitations such as selection bias and surgeon expertise. Additionally, the evolving landscape of pediatric MIS suggests the need for ongoing evaluation of LH techniques to optimize outcomes. This study contributes valuable insights into the surgical management of PIH, paving the way for informed clinical decisions tailored to individual patient needs and healthcare settings.
